# Extra Supplement.—The Nursing Mirror

**Published:** 1894-03-03

**Authors:** 


### "The Hospital" Nursing Mirror.

Being the Extra Nursing Supplement of "The Hospital" Newspaper.

[Contributions for this Supplement should be addressed to the Editor, The Hospital, 428, Strand, London, W.C., and should have the word "Nursing" plainly written in left-hand top corner of the envelope.]

### News from the Nursing World.

### THE PRINCESS MAY'S WARD.

There seems every prospect of the Royal Richmond Hospital possessing a fine children's ward of its own, and doubtless strenuous endeavours will be made to raise the additional funds necessary for the nurses quarters. Otherwise, as it was remarked at the recent meeting, until the executive part of the building is completed, "it would be impossible to use the children's ward as a children's ward." All our readers will cordially agree with us in hoping that the subscription list, which has already been so substantially supported, will be sufficiently enlarged to allow of the completion of the contemplated additions to the Royal Hospital. Mr. Charrington has promised to endow with £1,000 the Hilda May Cot when the ward is opened.

### PROBATIONERS IN CHARGE.

An interesting letter from a superintendent of nurses, which appears this week in another column, brings forward a subject which we shall be glad to have discussed. Charge nurses are considered by the writer as hindrances to the training of probationers. Such ought not to be the case, for the head of the ward, whether she be called staff nurse or sister, should consider the instruction of those under her as a duty second only in importance to the care of the patients. When she fails in either respect she is emphatically unfitted for her post. A capable woman teaches as she nurses, by example as well as precept, and we are much inclined to think that it is the fault of individuals and not of a system when charge nurses are found to be hindrances to the training of probationers. With regard to the nursing of a hospital by probationers only, it is probable that the patients would suffer considerably. A system which upholds the training of young nurses as paramount to all else, must assuredly be harmful to the sick. Thoroughly-trained charge nurses, who realise their duties to patients and probationers, are as esential to the proper working of a hospital as a qualified matron and competent ward sisters.

### WORKHOUSE NURSING ASSOCIATION.

The annual report of the Workhouse Nursing Association contains much interesting matter. One small fact will delight readers anxious for encouragement, for we learn the substitution of skilled for unskilled nursing has reduced the mortality in Halifax Union 37 per cent. in twelve months; and Miss Wilkie may rightly congratulate herself on the good work accomplished by herself and her staff. The objects of the Workhouse Infirmary Nursing Association, to which we wish continued success, are "to raise the standard of public opinion on the whole question of workhouse nursing; to secure the appointment of trained ladies as matrons in all separate infirmaries; and to train and supply nurses to workhouse infirmaries in London and the provinces.

### PROFITABLE PATCHWORK.

When the idea of a children's ward was mooted in the Essex and Colchester Hospital, a lady, whose husband had subscribed £50 to the cause, hit on a novel expedient for securing another donation. She had two quilts made of patchwork, each containing 140 squares, and every patch was added by a different person, who also gave a shilling to the children's ward. The trouble of this unique undertaking was cheerfully borne by Mrs. Holroyd, its promoter.

### HEALTHY ELY.

The annual meeting of the Ely District Nursing Association, held in the dispensary under the presidency of the Bishop of Ely, was well attended. The report was a satisfactory one, both as regards the work and financial position of the association. The sick poor seem greatly to appreciate the good nursing provided for them, and cases of bronchitis, cancer, rheumatic fever, pneumonia, dropsy, as well as casualties, have been successfully dealt with. Ely is to be congratulated on its freedom from epidemics, no less than on its well-organised district nursing, which is wisely supplemented by a nourishment fund.

### QUEEN'S NURSES AT GATESHEAD.

The Nursing Association at Gateshead laid its first annual report before the meeting of subscribers on February 22nd, and it proved to be a most satisfactory record of good work. The aim of those interested has been to establish the society on a firm, permanent basis, and they have already three Queen's nurses at work. Miss Peters, Inspector of the Jubilee Institute, has given the new affiliated association much assistance, and the nurses engaged by her advice at Gateshead have been much appreciated. They have a conveniently situated Home at 16, The Crescent, furnished by the committee and a few friends. The Superintendent, Miss Darrah, and the other two nurses are fully employed and highly appreciated by the sick poor. The first annual meeting was well attended, the Ex-Mayor being in the chair, supported by the Mayor and other influential people. Gateshead is certainly to be congratulated on the excellent beginning made by its district nursing.

### A QUALIFIED NURSE.

The Government Inspector has strongly recommended that a qualified nurse should be engaged for the sick wards at Upton-on-Severn Workhouse. As there are numerous bed-ridden patients, the need for a trained attendant is self-evident; but it is hardly likely that there will be many applicants for the £20 per annum, which the board decided to name in their advertisement as a suitable salary to offer to a qualified nurse.

### NURSES AT BIRMINGHAM.

A well-attended annual meeting of the Birmingham and Midland Counties Training Institute for

Nurses was recently held. Ten probationers have finished their training at the General Hospital, Birmingham, and at the Wolverhampton General Hospital. The Pension Fund has had an addition of £100 promised to it from the general account, and the condition of the institution appears to be altogether flourishing. The period of training has been increased, and although this adds to the outlay, the committee have wisely determined not to be content until their work is "as well done as it could be anywhere in the country."

### LIBERALITY AT LONDONDERRY.

A new house has been lately opened for the use of the Londonderry district nurses. It was given by Mrs. Tillie, and another house had been offered for the same purpose by Mrs. Bernard, but was not now needed, although the kindness was none the less appreciated. The society is prosperous, and is highly valued in Londonderry, and Bishop Alexander, who spoke on the occasion of the opening of the Home, hoped that Roman Catholics might soon be induced to be represented on the Board of the association, which was purely unsectarian in its work.

### FREE PASSES FOR NURSES.

In some places district nurses have free passes given them by the omnibus and tram companies, so that when "on duty" they are able to travel without any expense to themselves or their association. It is a small matter to the companies, but the favour is greatly appreciated, and it is a pity that it should not be more universal. At Dublin, the other day, fares were mentioned amongst the necessary items of district nurses' expenditure, but probably a suggestion to the tram companies that they should give free passes to the Queen's nurses would be agreed to without demur.

### QUEEN'S NURSES IN IRELAND.

The Archbishop of Dublin took the chair at the annual meeting of the Irish branch of the Queen's Jubilee Institute, and there was a large attendance of those interested in district nursing amongst the sick poor. St. Patrick's Home for Nurses, Stephen's Green, is the special title of the association which is affiliated with the Queen's Institute, and has a staff of eleven nurses. Three times as many cases were attended in 1893 as in 1891, and the association is still making rapid progress. Being absolutely unsectarian in character it is supported by all creeds, especially as it is now understood that it gladly supplements, and in no way aims at supplanting existing institutions and charities. Many applications have to be refused owing to the impossibility of the present limited staff undertaking all the calls made upon them. The needlework guild has rendered valuable aid by gifts of clothing, bed linen, and appliances. Hitherto the funds of the association have by no means increased in proportion to its work, and more annual subscribers are needed. The response to the special appeal made some two months ago was most liberal and satisfactory, but such worthy work as well-organised district nursing merits the substantial support of regular donations.

### ONLY HER DUE.

Eleven hundred patients, a hundred nurses, and seventy-five other women, are under the charge of the Matron at Birmingham Workhouse Infirmary. A visit to that beautiful institution shows very plainly that she is an exceptionally good organiser, and the Board of Guardians is well aware that they are fortunate indeed in having secured the services of Miss Gibson. Paying probationers are, to a certain extent, superseding other probationers, and therefore the management of the nursing branch is peculiarly economical, as well as efficient, and many more applications are received than can be entertained. At a recent meeting some members of the Board took exception to the proposed increase in the Matron's salary, which was a somewhat ungracious proceeding, as she consented to retain her present post by special request. The increase was eventually granted, but not as unanimously as might have been expected in a town deservedly noted for the liberality of its citizens.

### INDIA.

Any reliable information as to the work in Indian hospitals is always valuable, for comparatively little is known to the English public of the everyday progress out there. Recent correspondence in the *Army and Navy Gazette* shows pleasant evidence that some of the male native nurses are very good ones, and Corporal Ernest Callagher makes the very pertinent suggestion "that the nursing sisters be augmented, and the men of the Medical Staff Corps better instructed in the detail of ward work and the gentle handling of their charges." Surely these two points appeal to the common sense and consideration of all who are interested in the Indian nursing service.

### CHANGES AT ADDENBROOKE'S.

The nursing staff at Addenbrooke's Hospital is to have a new set of rules, and the form proposed was laid before a recent court of governors. The matter has been postponed to allow of due consideration, and before things are finally settled it is to be hoped that the committee will take counsel with the heads of some of the leading nurse training schools as to their systems. The additions to the old rules at Addenbrooke's appear rather to confine themselves to negatives. Such important matters as time off duty and recreation being, according to press reports, taken little account of.

### SHORT ITEMS.

The need for a fever hospital in Melbourne has again been pointed out. There is not a single bed to which a poor patient suffering from an infectious disease can be sent.—It is stated that the War Office has decided on the erection of a proper hospital in connection with the Royal Gunpowder Works at Waltham Abbey, on account of the numerous accidents which occur there.—Mrs. Cridland has been made nurse at Keynsham Workhouse at a salary of £30 per annum. —The Mayor (Dr. Wood) presided at the annual meeting of the Southport and Birkdale District Nursing Society last week. There was a large attendance on the occasion, and a good report was offered of the work of the four nurses.—Lord Grimthorpe presided at the opening of the Bath Homœopathic Nursing Home and Hospital on February 14th. The Local Government Board has sanctioned the proposal of the Guardians to appoint additional nurses for the infirmary.—A most successful smoking concert was recently given at Culley's Hotel, Barry Dock, the proceeds being devoted to the Barry District Nursing Association.

### On General Nursing.

By Rowland Humphreys, M.R.C.S., L.R.C.P.Lond.

### I.—MEASLES.

### An Infectious Disease.

Measles is an infectious disease almost confined to children over the age of six months, but which is occasionally seen amongst adults, especially in countries where it has not been previously known, as in the Faroe Islands in 1846.

### Its Characteristics.

It has certain peculiarities which distinguish it from other similar diseases; these are (1) the length of the incubation period; (2) the length of the prodromal period; (3) the severe catarrh which usually indicates the onset of the disease; (4) the peculiar rash; and (5) the great tendency to lung complications.

### Incubation.

The incubation period, the interval of time which elapses between the "catching" of the disease and its first definite symptom, is almost always fourteen days; indeed, if one says absolutely that the rash will appear at that date in a child who has just been exposed to the infection, one is almost certain to be right; but a few cases occur when the way in which the complaint has been acquired is not that in which it usually takes place, and then the incubation may be as short as eight days.

### Prodromal Period.

The prodromal period, the time which elapses between the first actual manifestation of the disease (usually a severe cold) and the appearance of the rash, which puts all "possible doubt whatever" to flight. This is three days, the rash coming out on the fourth.

These two characteristics distinguish it from scarlet fever, which is much shorter in respect to both periods, from German measles, where the incubation is similar, but the prodromal stage is almost absent, from small-pox in which, not uncommonly, a measly rash appears in the initial stage, but this is usually on the second day, and the characteristic back-ache and vomiting are present.

### Characteristic Catarrh.

The catarrh which precedes the rash is almost as characteristic as the rash itself. One often hears an old nurse say, "Well, I thought he had measles coming on, because he looked so heavy and had such a cold." The appearance of the child is, indeed, in most cases very striking. The eyes are heavy, flushed, and running, the eyelids swollen, the face puffy, and the whole attitude expresses a feeling of disinclination to move or play. The toys lie in front of him, but their presence seems merely to irritate the little patient from his very inability to enjoy them. There is a free discharge from the nose; there is a cough, and probably some amount of wheezing; and often some diarrhœa or looseness of the bowels, all indicative of a severe general catarrh. If the throat is now examined a speckled, or even generally injected, appearance of the soft palate will be seen, and very commonly thin opaque white patches on the gums or inside of the lips are present. The glands at the angles of the jaws in the neck are usually enlarged, and the tonsils are swollen.

### The Rash.

The next symptom which is peculiar to the complaint is the rash. That this, as already mentioned, appears on the fourth day, is practically true, though the time may be a little shorter. In appearance it is very like a collection of flea-bites, but without the central puncture, though, of course, the two rashes may be mixed together. The course of the disease and the other symptons will soon explain matters. The rash appears first at the roots of the hair, and thence spreads downwards over forehead, face, and body, to the arms and legs, reaching these last at about the third day. It comes out in crops, of which each crop lasts twenty-four hours. Each spot is separated from its fellows, except where they have become confluent as often happens on the face, by an area of clear skin, and each new development of spots seems to form an incomplete ring round a predecessor; thus the first spots are scattered about in no particular order, the next ones come out in circles round these, the ones which follow in curves round the last arrivals, and so on, each fading as its successors make their appearance. Thus the spots run in curves, each spot separate and distinct. There is no other infectious disease which has a rash which is thus distributed, and which is accompanied by the other characteristics but the complaint under discussion. The rash leaves little brown stains to indicate where the spots have been, and these only disappear when the skin scales off during the second or third week. An antiseptic inunction may be used during this period.

These, then, are the characteristic early symptoms of the complaint.

### Special Dangers.

Having seen that a severe cold is the earliest symptom, it will be readily understood that with a complaint in which *the* especial danger lies in the tendency, as will be shortly seen, to lung complications, it is as well to confine the patient to one well-ventilated, warm room; and in cold or damp weather it is a good plan to put on the steam kettle with some antiseptic, such as carbolic, creolin, or eucalyptol in it. This is also the most infectious stage of the disease, so isolation should at once be carried out to its full extent, and all children who have been in contact with the patient should have their clothes changed and disinfected, and be them selves isolated from others for the full incubation period, *i.e.*, fourteen days. The reason why adults so seldom take the disease is that they have almost invariably had it in their youth. One attack nearly always protects from a second, and in many cases in which a second attack is said to have occurred soon after the first one, there has been an error in the diagnosis due to the tendency of the rash, which does not in itself constitute the disease, to reappear with any slight irritation of the skin. Thus, in a child which the author saw in consultation, he was told that the patient had had measles some little time before, and had had a second attack of the same disease some months afterwards, and that it was now suffering from a peculiar rash which was like a mixture of scarlet fever and measles. There was a distinct scarlatina rash, and mixed with it was another form of rash just like that of measles. However, there was no catarrh, and the other symptoms pointed to the former rather than to the latter complaint, and directions were accordingly given. This diagnosis the friends disagreed with, and no precautions were taken to isolate the child, the result being that five other children in the house took the disease in the undoubted scarlet fever form, from which two of them nearly lost their lives. This brings us to another point: the very casual way in which measles is, as a rule, treated. The mother says, "Oh! I don't think it is any good to isolate this child; the others are sure to get it." She does not know that the disease is carried out of the house on her clothes, and that in taking no precautions to save her own children she is exposing her neighbours to considerable risk, which may cost some of them their lives.

### Presentations.

The nursing staff of the Sheffield General Infirmary recently presented Miss Rickards, their Matron, with a handsomely-bound volume of Longfellow's Poems and a beautiful piece of china.

### The Beginnings of Insanity.

(BY A MENTAL NURSE.)

I SUPPOSE that most people know what they mean, or think they do, when they speak of insanity. It is only those who have lived with the insane, and have observed with seeing eyes the strange phenomena of insanity, who know how difficult a thing it is to define with words; and it is only those who have closely watched the early stages of brain disease, and have marked the slow, gradual, sometimes imperceptible, advances by which it saps the foundations of reason and intellect, who realise how uncertain is the border line between sanity and insanity, and how easily, at any time, any one of us may overpass it.

My own experience is, that very crude ideas on the subject of insanity exist among people of ordinary culture and intelligence. One is, that all lunatics are, at all times, raving and dangerous wild beasts. This idea is hardly worth correction. Another is, that all lunatics are monomaniacs, and perfectly sensible on all subjects save one; and yet a third is, that all lunatics are idiots. It is hardly necessary to say that monomania is only one form of lunacy, and that lunacy is quite a distinct thing from idiocy. Such distinctions are easily explained, but false impressions on a subject which is little understood are not so easily corrected; and I think myself that if we had more knowledge of the beginnings of insanity —those apparently trifling, unimportant beginnings, which sometimes lead to such disastrous endings—we should also have a better understanding of what insanity really is, and what our responsibilities may be with regard to it.

By the beginnings of insanity I do not exactly mean the causes of it. A list of those can be seen by anyone who looks in the annual report of any asylum. Heredity, drink, accident, overwork, loss of money, and so forth. But even where the disease is traced to a direct cause, such as drink or overwork, in nine cases out of ten there is an hereditary predisposition; and this leads us to the question, how long has it been hereditary? Where did the disease have its insidious beginning, and through how many generations has it descended to its present victim? Somewhere in those past generations, in some individual, long dead and forgotten, the taint must have had its first beginning; and if so, somewhere in the present day, in individuals now living, insanity must in some way have its first beginning, to be transmitted by them—terrible thought!—to an unknown number of future generations.

It will help us to an understanding of these beginnings of insanity if we consider the means by which we try to cure those cases which are deemed curable in asylums. It has more than once been said to me, by those who knew something of the daily routine of an asylum, that nothing seems to be done to cure the patients, or to make them better. The treatment is not apparent, as in a hospital; there is comparatively little medicine given, and at first it is hard to understand that discipline, routine, strict control, and constant occupation are by far the best remedies that are at present known to us for those whose disease is, practically, that they have lost their self-control.

How did they lose their self-control?

In the first place we must remember that self-control with most of us means simply an outward self-control, which is imposed by the necessities of social intercourse, and the permanent loss of which is generally supposed to constitute insanity; but this loss is really but the outward sign of the inward loss of true self-control, by which I mean the power of keeping a constant check upon idle and wicked thoughts and unruly emotions. Some people have so little of this true self-control that a slight cause will be sufficient to deprive them of it; others have acquired so much that it will bear a severe strain before giving way.

If we honestly consider how much, or rather how little, of real inward self-restraint we practice in our daily lives, we shall perhaps realise how perilously near most of us stand to the edge of the precipice; before we are aware our feet may slide, and our fall may be beyond remedy.

Self-indulgence has many forms, and the most dangerous to a sound condition of mind are sometimes the least noticeable to outsiders. Such forms of excess as drink may be checked by outside influence; but we may have the habit of indulging mean and unworthy thoughts and wishes, useless dreams, or jealous and suspicious fancies, without anyone being aware of it except ourselves, until the time comes when some severe shock, or strain upon the nerves, shatters the outworks of conventional restraint; within there is no stronghold to fall back upon; we have never striven to check the inward current of our thoughts; now we have lost the power to check their outward expression; we utter them aloud, we act upon them, and we are rightly looked upon as insane.

But a person in this condition has not become insane all at once, nor even in a few weeks or months, as is often supposed. Through many years, perhaps a lifetime, of mental self-indulgence, he has gradually weakened his reason and his intellect; but it is only now that circumstances have made his growing insanity apparent to others, perhaps also inconvenient and dangerous. Of course, the circumstances may never occur, and the man may go to his grave without ever being reckoned otherwise than sane; but let us remember that we all carry these seeds of insanity within ourselves, and that no one of us can dare to say that he shall be the one to escape.

An hereditary predisposition, if we know of it, should by no means serve as an excuse, but as a warning. The mischief that has been done by past generations may yet be undone by future ones; and if the curse has fallen upon ourselves, our efforts may remove it from the heads of those who shall come after us. And in this respect a great responsibility devolves upon those who have the training of children, whether their own or those of others. It is unspeakably cruel, in any case, to leave children undisciplined and uncorrected, and therefore unprepared for the trials of life; but the cruelty is aggravated in those cases where insanity is known to exist in the family. It is probable, and only natural, that children with insane parents or relatives should be particularly excitable, nervous, and wayward in disposition. For this very reason they are frequently indulged and given way to, until they become perfectly unmanageable, instead of being kept under that firm and constant control which is more necessary even for them than for other children. But the outside world will have no such consideration for their failings. They must encounter the ups and downs of life, and take their chance with the rest; and the undisciplined brain, ill balanced by nature, and unfortified by education against the assaults of temptation, anxiety, and disappointment, gives way under the ordinary stress of existence, and the spoilt child too often ends his or her career in an asylum for the insane.

I believe that in the majority of cases, if not in all, self-indulgence, either in the individual or in some of his ancestors, is the beginning of insanity. An undirected imagination, unbridled thoughts, hours wasted in useless day-dreams, sensitiveness encouraged to the pitch of absurdity, morbid suspicions and fancies continually brooded over, and allowed to take a firm hold of the mind, affections, right and natural in themselves, but extravagantly and blindly indulged; all these in their first beginnings are but small things, unnoticed or treated with contempt. Yet the ruined lives, the wasted powers, the disappointed hopes, the heartrending misery, which are the endings of these beginnings, no man may reckon.

Shall we wrestle with ourselves, if perchance we may avert the curse, or shall we dream on, not caring, if only we ourselves escape, that it fall on our children, and on our children's children?

### Lectures at St. George's Hospital.

HISTORY OF THE HOSPITAL.—*Continued.*

Mr. Dent's second lecture on this subject dealt with an especially interesting chapter in nursing history, showing the almost incredible advances which have been made since the building of the new hospital, which was finished in 1834, exactly one hundred years after the opening of the original institution.

In the early days of the present century hospitals did not stand high in the public estimation, and no very sharp distinction was made between the inmates of a hospital and the inmates of a workhouse. Those who nursed in hospitals did so, many of them, from a very high motive, but, as a rule, from a personal one. They selected work which was considered degrading and horrible as a penance and a mortification to themselves. The scientific spirit simply had no existence, except among the medical officers, and the medical officers never thought of encouraging this spirit in the nurses who worked for them. The philanthropy of such people as John Howard and Elizabeth Fry was really of a higher character than can now be quite realised. Disease was then looked upon as loathsome and horrible, and to be avoided. The perpetual glare of criticism was not then directed on charitable institutions by the Press, and compulsory improvement was not a necessity of their existence as now, when the words "develop and improve or die" might be written on hospital walls. In 1835 it becomes evident that the engagement of nurses has passed into the Matron's hands, as now it is first mentioned that the nurses are introduced by her to the Weekly Board. The wages by this time have risen considerably, as we find that the head nurses are now receiving £21 a year, and there is evidence to show that there was no dearth of nurses. In 1834 the nursing staff numbered 36, the beds being 300, and from this time there was almost a yearly increase, till in 1845 the figures reach 45 the proportion then, however, being only one nurse to seven or eight beds.

The first probationer was received in 1844, and the circumstances of her reception are interesting. A letter is extant from Miss Elizabeth Rogers, the head of the Society of Nursing Sisters, to the authorities of St. George's Hospital, stating that the society had long desired the privilege of having a nurse trained at the hospital, and asking for one of the sisters to be admitted, the society allowing her board wages during the probation of two months at the hospital. Hitherto the London and Guy's were the only hospitals to accept probationers, and St. George's was thus the third London hospital to adopt the system. Mr. Dent has consulted one or two eminent authorities—Sir James Paget, Mr. J. Birkett, and Mr. Charles Hawkins—with reference to nursing matters fifty or sixty years ago, and they used almost the same words in describing the nurses of their various hospitals; in fact, what might be said of one could be said of all. Fifty or sixty years ago! It does not seem so long, yet it must be remembered that those were days when anæsthetics had never been used in surgery, when such operations as ovariotomy were absolutely unknown, when sanitary science was not, and typhus, under the name of gaol and other fevers, ran riot. There was then, as there is now, true devotion and genuine sympathy, but the efforts were misdirected, and after treatment was to a very great extent neglected in hospital wards. Sir James Paget describes the nurses as having "no art, and being quite unable to keep a chart or take a temperature; many of them were rough, dull, unobservant, and of the best it could only be said that they were kind and careful in doing what they were told to do." The admission of ladies would not have been possible, and nothing but mischief would have been predicted of such an experiment. The arrangements for the meals of the nursing staff were uncomfortable in the extreme. The meals were taken in the wards as best might be, and Mr. Charles Hawkins well remembers seeing the nurses toasting sausages for their dinners at the ward fires.

The Crimean War found us utterly unprepared for such an outbreak in the matter of nursing. The supply of nurses with any proper degree of training was very limited, but many women applied for training in order to go out to the seat of war. Whatever politicians and others may say of the Crimean War, at least it is directly responsible for one good—it brought forth Florence Nightingale. The horrors of those hospitals in the East, crowded with the sick and wounded, caused Florence Nightingale to offer to go out and organise the nursing at Scutari, and it must not be forgotten that as prominent as she in the whole matter was Sydney Herbert, whose name is now somewhat apt to be forgotten. And among the nurses sent out to the East was one from St. George's Hospital, Sarah Tapp, whom, it is interesting to know, afterwards returned to the service of the hospital and retired, not so very long ago, with a pension, and is still alive. The little band of nurses reached the hospital on the eve of Inkerman, and no words can adequately describe the horrors of that time. The sick and wounded had to be transported first down to Balaclava, and then across the Black Sea, the mortality on the way being something appalling, while "Scutari, the longed-for haven, was crammed with misery, overflowing with despair." In the hospital the patients were ranged in two rows down the wards, feet to feet, fronting each another victim of war, or starvation or pestilence, and every day nearly a hundred deaths took place, by far the greater number being from cholera, dysentery, erysipelas, or other preventible diseases.

And the impetus given to nurses by the example and writings of Florence Nightingale has had lasting effect. It is not too much to say that she originated the present type of nurse. In a few words the change in nursing matters can be described:—It was a trade, and a bad trade; it is now a profession, and a fine profession. Although St. George's Hospital, said Mr. Dent, is by some considered to be behind the times, it is worth recording that the anthorities of this hospital were the first to offer to Florence Nightingale the compliment of electing her as an hon. life governor, thus being the first metropolitan hospital to place a woman's name among the lists of governors.

And now improvement spread rapidly. A uniform was adopted, and with it came the sense that nurses represent their hospital for good or the reverse, and that its credit is largely delivered into their hands. Dining and sitting rooms are provided by degrees, and better sleeping accommodation, and the staff is largely added to. The change is more apparent in the position of the assistant nurses than in that of the head nurses, and scrubbers and ward-maids are employed for the rough work hitherto done by the assistant nurses and night nurses. And so the history of nursing comes down to the present day, when there is yet a question of still further lightening the work by having the nursing divided into three shifts, though whether this is ever carried out will depend upon the question of pounds, shillings, and pence, inasmuch as improvements in a hospital are just exactly dependent on the funds supplied by the public for such purposes.

Mr. Dent concluded this brief sketch of the history of St. George's Hospital by hoping he might have been able to arouse in the hearts of his audience some of the affection which he felt himself for this great institution, assuring them that everybody there was as much interested in the making of good women into good nurses as in the turning of students into good doctors. Some nurses said after they had left the hospital that they had "learnt nothing," but the opportunities were magnificent if advantage were taken of them, and only to learn *how* to learn was to know a great deal. The world would not creak so much if people devoted less time to complaining of things as they ought to be and are not, and more to making the best of things as they are.

### Women's Work.

### HIGH SCHOOL TEACHERS.

(By a Late Teacher.)

High-School teaching is, as a rule, taken up by those who have themselves received a High-School education, and who have, therefore, some experience of the work and methods. Very often the girl who intends to be a mistress will pass from pupil into pupil teacher (or, as it is generally called, student mistress), and while working up for her examinations and keeping in, to a certain extent, with the ordinary school course, she is expected to assist in instructing the younger children. This is no light work, altogether, for a girl of seventeen or so.

Others, again, go straight from school to college, and after spending the necessary time there start as full-fledged assistant-mistresses—not, as a rule, on a very magnificent salary, for, considering the amount of work it involves and the trouble and expense of the preliminary training, High-School teaching, in its earlier stages at least, is not well paid. Efforts have been made by co-operation to raise the commencing salary of a thoroughly qualified assistant-mistress to £100 per annum; as yet, unfortunately, without success.

This sum does not seem very exhorbitant, and when board and lodging have been paid for, it does not leave a very large margin to put by; for it must be remembered that the time during which a High-School teacher can work is comparatively short. As time goes on the younger workers elbow her out of the ranks. Head mistress-ships, of course, there are, which for the most part are very well paid; but it stands to reason that these are not attainable by all. "I have made up my mind to go to Sierra Leone in a few years' time," a High-School mistress was heard to say to another, half laughing, half serious; "you see, I shall soon be getting old, and then what am I to do for work? Now, in Sierra Leone everybody dies of fever!"

Of course, this is an intensely pessimistic view of the situation, but it is just as well for those who are thinking of entering the profession to remember that the years of their labour and gain may be few, and the labour while it lasts is hard.

To interest the minds and keep quiet the bodies of a class of, may be, thirty to forty girls, the mistress must prepare beforehand almost every word of the lesson she has to give. She must know exactly what she is going to do and say, so as to be able to give her attention to what is going on about her, as well as to her books. She must be prepared, too, to answer questions of all sorts and kinds, such as the infant mind loves to perplex its mentor with. Then, when school is over, there will be the piles of exercise books, of arithmetic papers or of maps to be carried away and corrected, as well as the next day's lesson to prepare.

There are disappointments, too, in the work, and especially for the young teacher with theories of her own and a burning desire to put them into practice. Tied down by cut and dried regulations, she sometimes gets apathetic in her work, and drags her pupils, without interest, through the text-books prescribed by the authorities who regulate the affairs of the Cambridge Local Examinations. Of these examinations it is enough to say that, by the premium they put upon superficial knowledge and unoriginality, they are doing more harm amongst the middle classes than will easily be undone.

There is another drawback in too many of our schools which the rising generation of teachers must help to remove. Anyone with the least knowledge of the rudimentary laws of health must be horrified at the stooping shoulders and sickly complexions which too often disfigure girls in the schoolroom. If this matter is mentioned to a schoolmistress, she will probably say that "the children have so many hours drilling a week," almost with the air of asking what more anyone could want. But, after all, drilling is not a panacea for every ill (and they are many) produced by overwork and lack of exercise. The anæmic girl, her brain tired by "prep." and classes, goes back to her school-room, after half-an-hour's drill and dumb-bells, with her body also tired, and perhaps gets a scolding for lounging over her desk iustead of sitting up with a straight back.

The fault lies not so much with any individual mistress as in the system under which the girls are worked, always at full pressure, and the slower a girl is naturally, the harder becomes the strain to keep up with her fellows and the worse the effect upon her health. The evil is a great one, and it will continue as long as the system of sacrificing the body to the fancied advancement of the brain is carried on. If High-School mistresses would but realise this, and band themselves honestly together to fight the enemy, there is hardly any limit to the good they might achieve.

Sir James Crichton-Browne has spoken strongly concerning the unsatisfactory state of health of the pupils at High-Schools, and unprejudiced witnesses will surely bear him out. The evil once recognised, it remains but to root it out successfully.

### Novelties for Nurses.

### AN IDEAL DRESS MATERIAL.

(Messrs. Garrould, Edgware Road.)

Messrs. Garrould have sent us samples of the "Harrisburg' all wool beige, which meets the long-felt difficulty experienced by nurses of finding a woollen material which neither shrinks nor loses colour in washing. It is really an ideal material for the use of nurses, and cannot fail to find patronage generally when its advantages are known. The colour is a soft grey. The material is firm, light, warm, and soft. A severe test under soap and soda, leaves the tint unchanged, and the substance close and soft. Lastly, it is a most inexpensive material, as, whilst possessing all these qualities to recommend it, the price is only 2s. a yard, the width being forty-five inches. Nurses cannot do better than send for patterns.

### USEFUL JEWELLERY.

(J. N. Masters, Rye, Sussex.)

Nurses are seldom lovers of jewellery, nor, indeed, have they much opportunity for wearing it. Nevertheless, watches and brooches are popular necessities. Mr. Masters, of Rye, has a wonderfully nice stock of both of these at very low prices. His jewellery of all descriptions is of excellent quality, and as the cost of establishments such as his is far less than one in the metropolis, he undertakes to sell at 20 per cent. lower than London prices. He makes a speciality of very attractive little watches. The shell-back watches have a seconds circle for accurate timing, and are offered to nurses on special terms. The shell pattern is novel and pretty. Neat little chains can be procured in silver as low as six shillings, and in gold for twenty-five shillings. It is a great advantage to purchasers that they are able to have nearly all patterns in silver and two qualities of gold. The catalogue would prove useful for reference. Full particulars of all kinds of jewellery are given, and it is profusely illustrated.

### Minor Appointments.

Kensington Infirmary, Marloes Road. — Miss Nina Allison has been made Sister at this infirmary. She was trained at the Sussex County Hospital, afterwards joined the York Home for Nurses, was Ward Nurse at St. Saviour's Infirmary, East Dulwich, and Staff Nurse at the City of London Hospital for Diseases of the Chest.

Kidderminster Infirmary.—Miss B. Jones has been appointed Sister to the Kidderminster Infirmary and Children's Hospital. Miss Jones was trained at the Westminster Hospital. Since then she has held the post of Night Superintendent at the Blackburn Infirmary. For two years she was Sister-in-Charge of the General Hospital, Barbadoes, and has also held the post of Sister in the Chelsea Hospital for Women.

### Health Resorts for Consumptive Patients.

It is not more than a few decades ago that a sojourn in Southern Europe was looked upon as about the only likely cure for consumption, although innumerable sufferers from this terrible affection never again returned to their own country from such a visit. This was due to several causes; in many instances the journey was only undertaken when the affection was already too far advanced, and it had not yet been realised that change of air was only one stage in the treatment of consumption, a stage certainly which is of great importance, but which cannot under any circumstances compensate for a rational, dietetic, and medical treatment.

The prospects of recovery for persons suffering from consumption are brighter now, and this has been brought about, more than by anything else, by the erection of regular and real health resorts or sanatoriums, the oldest, and for a long period the best-known, of which was the institution erected by Dr. Brehmer at Görbersdorf in Silesia. It dates from 1859, and the principle upon which it was conducted was quite a new one. Dr. Brehmer thought that a sojourn in an elevated spot, protected against wind, and a fairly uniform climate were bound to have a strengthening effect upon the system by stimulating the functions of the heart, and in practice this theory has apparently proved correct, as the Görbersdorf institution has given most satisfactory results in manifold cases. The theory has, however, not really been proved, and as the good results were more to be accounted for by the careful treatment than by the mountain climate, a number of similar resorts have been erected at places where the climatic conditions have been totally different.

The health resorts for consumptive patients can be divided into two classes, the open and the closed sanatorium. The first comprise many along the Mediterranean coast. The Riviera has quite a string of climatic stations, extending from Cannes to the neighbourhood of Pisa, Mentone being the most important; but there is, more or less, the same objection to all of them—that they are too much frequented by healthy persons. Consumptive persons experience a considerable difficulty in finding that quietness and that thoroughly systematic manner of living which suits them best. In addition to this, the climate, although possessing many favourable qualities, also has its distinct drawbacks. The dew fall is very heavy, and the mistrale, a violent, very dry, and cold northerly wind, which sweeps down the Rhone Valley through the Riviera to the vicinity of San Remo, is particularly dangerous. In several places a few days' dry weather is also conducive of a considerable amount of dust, which is, of course, very objectionable. Nor is, finally, the weather during the winter so warm and uniform as is generally believed.

The health resorts on the borders of the north Italian lakes have, on the whole, a somewhat colder, but more uniform, climate. Many of them possess exceptionally good natural conditions for consumptive patients, but they are in other respects so primitive that they cannot be recommended without reserve, although they are being extensively advertised—like Lugano, Pallanza, Locorus, Riva, &c. The same applies, in a less degree, to several resorts in Southern Switzerland, which are evidently more intended for exploiting the tourist than giving the consumptive patient a chance of benefitting from the excellent natural and climatic conditions. There is only one of these places which is deserving of praise, viz., Arco, in South Tyrol, to the north of the lake of Garda, which enjoys a warm and uniform climate, without wind, charming environs, and a sanatorium of limited dimensions—an advantage which cannot be too highly estimated. Montreux, which really comprises a whole series of small towns on the north-eastern border of the Lake of Geneva, and formerly enjoyed a considerable reputation, is now too built up and too frequented by tourists to be desirable for patients.

Among all the open resorts the place of honour must be given to Meran in Southern Tyrol, where more has been done for the comfort of the patients than in any other place, and where, besides, the natural conditions are exceptionally favourable. Meran is situated in a valley, which to the west, the north, and the east is surrounded by high mountains, whilst from the south there is access for currents of warm air. The height above the level of the sea is some 1,000—1,500 feet, a position which entails moderate mountain climate, with a dry, light atmosphere. The summer is a little too warm, but during the autumn and the earlier part of the winter up till February the weather is generally excellent. In addition to this the surroundings are beautiful and the whole treatment of the patients is splendidly arranged, so it is not to be wondered at that Meran of late years has been much frequented. The same drawback, however, attaches to Meran as to other open resorts—the season of the year must be consulted; one has to go there as early in the autumn as possible in order to leave as early in the spring as possible, not to return home, however, but to go to some colder mountain resort.

The Alpine sanatorias are of a very different type, but only one of these deserves special mention, viz., Davos, in the Canton of Graubündten, which was only discovered as a resort some thirty years ago, and yet receives as much as 10,000 visitors per annum, of which number about 1,500, healthy and patients, winter there. Davos was originally only a summer resort, like Görbersdorf, but it only took the patients a few seasons to find out that the place suited them so well that they stopped all the winter. It was then observed that the winter was the best part of the year for the consumptive patients, although it is by no means mild. From the end of October to the beginning of April the average temperature of the day is below freezing point, and during that period the earth is covered with a layer of snow, which is often several feet deep, and which does away with the dust and organic processes. The coldest months are December (÷ 5·5 Centigrade), January (÷ 7·4 Centigrade), and February (÷ 4·2 Centigrade); and during these months it is quite a usual thing that the thermometer during the night falls below ÷ 20 Centigrade. This cold weather does not, however, prevent the patients from being in the open air, for the noonday temperature often approaches, or even rises above, freezing point, and on account of the thinness of the atmosphere the direct force of the sunbeams is so great that it is possible to sit still for hours in a sunny place, and even feel tempted to take off extra wraps. Also during the evenings it is possible for the patients to be in the open, sitting or lying down, well wrapped up, as it is generally very calm; and there are even instances of patients having been lying out in a temperature of ÷ 26 Centigrade without having suffered any harm.

A few years ago a sanatorium for consumptive patients was built at Davos—a first-class institution, built in full accordance with the most approved scientific principles. The patients are there subjected to a severe discipline, and not allowed to join in the wintry sports and diversions, and the results of this closed resort have also been still better than those experienced at Davos prior to its erection.

The Davos Sanatorium is arranged on the same principles as those at Görbersdorf and Falkenstein, although the climate is much milder at the two latter places, the position of which is also much less elevated than Davos, which lies some 5,000 feet above sea level. They have large rooms where the patients spend the greater part of the day in the open air in a reclining position, but protected against wind and rain, and this is the leading factor in the treatment, coupled with plenty of nourishing food. Neither at the two above mentioned places, nor at the new sanatorium Hohen-Honnef, is the elevation above sea level important, and more especially the latter place, which is situated in the Siebengebirge, close to the Rhine, does not possess any very distinct climatic peculiarities.

☞ For The Muses' Looking Glass, Novelties for Nurses, Everybody's Opinion, &c., see page ccxix. et seq.

### Our Melbourne Letter.

The beginning of the New Year was unpleasantly marked in Melbourne by an alarming increase in the number of typhoid cases. In 1893, during the first fortnight of January, the official returns for the whole colony gave 78 as against 142 in the corresponding weeks of 1894. An increase in cases of typhoid during the months of January, February, and March is usual, that being the hottest season of the year; and, in fact, these are often called "the typhoid months," though, as a matter of fact, the disease has been endemic for many years; but this sudden increase is somewhat of a puzzle. At the last meeting of the Board of Public Health the matter was taken into consideration, but no feasible explanation was forthcoming. Dr. D. A. Gresswell, medical inspector, animadverted upon the facts that dairymen are slow to learn cleanliness, and that the water supply of Melbourne is liable to be contaminated through faulty fire-plugs. A negative sort of satisfaction can be gathered from the figure returns in that, while the number of cases for the whole colony this year is nearly double that of last year, the number of fatal cases, nine only, remain the same. During the fortnight specified the cases of diphtheria showed a marked decrease, and scarlet fever a slight increase. The Melbourne Hospital Committee, at one of their fortnightly meetings, warmly debated the question of appointing a special anæsthetist. A death under chloroform occurred there recently, and at the inquest the City Coroner, Dr. Youl, as on several previous occasions, strongly urged the necessity of the appointment. The proposal met with strenuous opposition from other members of the committee. The subject is to come up again for discussion. It is understood that the medical staff, who were formerly opposed to the appointment of a special anæsthetist, have changed their views. The share of the Hospital Sunday Fund received by the Melbourne Hospital this year amounts only to the sum of £1,150, as against £2,100 last year. The under treasurer has written to the various hospitals and charitable institutions notifying them that in future they will be required to pay for all railway passes. In the past prosperous times, when a patient was transferred from one hospital to another, or a poor old man sent to a benevolent asylum, the Government always, on application, granted a free railway pass.

### Notes and Queries.

### Queries.

(12) *Australia.*—Can any of your correspondents tell two trained nurses what prospects of success they would have in Australia?—*Nurse Leicester.*

(13) *Colonial Nursing.*—Where can I get information about colonial hospitals?—*Nurse Janet.*

(14) *Sick Pay.*—Can anyone give the address of a society into which a lady earning her own living can pay a premium to receive sick pay?—M. R.

### Answers.

(12) *Australia* (*Nurse Leicester*).—You would find The Hospital a most useful book of reference if you kept the bound volumes by you. There has been correspondence on this subject before. The training schools in Australia are able to supply nurses now, and there are already so many English and Americans out there that, unless very well introduced, private nurses going out on their own account would not have much prospect of success. We believe the doctors appreciate English training, but there is a good deal of jealousy felt of them by the colonists.

(13) *Colonial Nursing* (*Nurse Janet*).—As regards these hospitals, you cannot do better than get Burdett's "Hospital Annual" (new edition). See answer No. 12. You had better consult the Manager, at The Hospital Office, 428, Strand, about bound volumes of the journal.

(14) *Sick Pay* (*M. R.*).—You do not say what profession you follow, and whether a provident club is what you mean. Please write more fully.

### For Reading to the Sick.

### SANCTIFICATION.

**Motto.**

Sickness is a fire which purges all rust if you are iron, and purifies you if you are gold.—*Ancient Father.*

**Verses.**

. . . These severe afflictions
Not from the ground arise,
But oftentimes celestial benedictions
Assume this dark disguise.—*Longfellow.*

Hast thou gone sadly through a dreary night,
And found no light,
No guide, no star, to cheer thee through the plain,
No friend save pain?
Wait, and thy soul shall see, when most forlorn,
Rise a new morn.—*A. Proctor.*

But if, impatient, thou let slip thy cross,
Thou wilt not find it in this world again,
Nor in another; here, and here alone,
Is given thee to suffer for God's sake.
In other worlds we shall more perfectly
Serve Him, and love Him, praise Him, work for Him.
. . . . . . .
To suffer is our appointment here.
Canst thou not suffer then one hour—or two?
If He should call thee from thy cross to-day,
Saying it is finished!—that hard cross of thine
From which thou prayest for deliverance,
Thinkest thou not some passion of regret
Would overcome thee? Thou wouldst say "So soon?"
Let me go back and suffer yet awhile
More patiently; "I have not yet praised God."
And He might answer to thee: "Never more;
All pain is done with." . . . . . .
. . . . . Let us take heed in time
That God may now be glorified in us;
And while we suffer, let us set our souls
To suffer perfectly: since this alone,
The suffering which is this world's special grace,
May here be perfected and left behind.
—*H. Hamilton King.*

**Reading.**

The thought that by our sufferings we are being sanctified, is one which is too rarely dwelt upon . . . but it would be a help to many to look upon the time of suffering as a necessity, and that without it we cannot be purified. . . . Suffering is the discipline of God sent to mould us. . . .

You must take care that the infirmity of your body does not infect your soul, and do not think that your sufferings or your weakness exempt you entirely from your religious duties; or that because of indisposition you may dispense with making progress in the spiritual life. God does not require of you the work you would gladly do for Him if you were in good health, but He does require that you should serve Him even in your weakest hours. Lift up your hearts to Him when you cannot collect your wandering thoughts—utter some words of ejaculatory prayer when you feel powerless to make any effort, when your weakness and suffering seem to be increasing. . . .

Your sufferings can be offered to God. Patiently borne, cheerfully accepted, they will prove to be a sure means of your sanctification. To be very quiet concerning your troubles is well pleasing to God. He rejoices to see you patiently bearing suffering known to Him alone. He cannot err as to the amount of suffering necessary for you to bear. It has been truly said that one single day of illness, borne with patience and resignation may advance us further in virtue, obtain for us greater grace, and make us more agreeable to God, than a week or a month of health. In your weakened condition you need strength; but God will give you all you need; for "His strength is made perfect in weakness." . . . Resolve then, that though you do not understand the dicipline now, you will bear your sufferings with patience, gentleness, and submission, for some day you will bless God for them, when you realise how through them you were sanctified.—*M. E. Granger.*

### The Muses' Looking Glass.

BONA FIDE FACTS ABOUT BONES.

We have more than two hundred bones in all,
Plenty to break, should we happen to fall;
But should this occur, don't go to a bone-setter,
A qualified doctor will mend you much better.
These bones, says Mr. Huxley the sage,
Vary at different times of age.
But not e'en the humblest seems to lack
Thirty-three Vertebræ down his back.
Now, twenty-four of these said bones
Are really happy in their homes;
But then come five, who say 'tis humdrum
To keep like this, let's join the Sacrum.
The next four say, if them's your tricks,
We'll join the club, that's named the Coccyx.
Now if this is not one of old Huxley's fibs,
Every one numbers twenty-four Ribs;
Twelve on each side, and all neatly connected,
With Cartilage to Breastbone clearly detected.
In the girdle which binds the shoulders around
Two sizeable bones are very soon found.
Scapula and Clavicle, very grand names,
But to say them too often might turn any brains.
The Pelvis, to which both the legs hold on,
Consists of two bones like the name of a song.
Oh "Ossa innominata" sounds grand sung,
These divide into three while you're very young.
And if I do not tell you at once
The names of these three, you will say I'm a dunce.
So Pubis, Ischium, and Ilium they are,
They sound quite absurd, but blame their mamma;
For she being young, and only a woman,
Said these very queer names were so "sweetly uncommon."
If you suffer from rheumatism in either arm,
Thirty bones there may well give you alarm;
Thirty bones in each leg too has every feller,
By counting the knee-cap, or Patella.
Before passing on in quite easy stages,
These bones bind together by Cartilages.
And where they play freely, one over the other,
A Cartilage coat prevents all bother.
And should you wish to be very particular,
This Cartilage goes by the name of Articular.
These by a delicate membrane lined,
Secrete oily fluid, isn't that kind?
This liquid is always called Synovial,
If that runs short, you won't feel jovial.
As you may have already thought,
The feet are the surfaces of support.
But should the muscles of Calf not contract,
You'd tumble forward, that's a fact.
But this act would bend the leg one way,
So the front of the Thigh muscles come into play.
E'en then, though you try with all your might,
Without help from the Back, you can't stand upright.
To be governed by legs is hard, but depend on,
You'd be certainly worse, if they wern't there, to stand on.
Now before I leave this subject, I beg
You will learn the names of the bones in your leg.
The Femur, which lasts from the Thigh to the Knee,
Is a most useful bone, as of course you all see.
And then too, below the knee we find,
A Tibia in front, and Fibula behind.
The Tarsus, and Metatarsus, bones come next,
I can't find a rhyme, so you mustn't be vex't.
Then last, and least, come Phalanges or Digits,
These covered with chilblains, will soon give you fidgits.
Before going on, there won't be much harm,
In saying the names of the bones in your Arm.
Like the bones in the leg, they are really not numerous,
But from Shoulder to Elbow, you'll find one, called Humerus.
Then come Radius, and Ulna, from Elbow to Wrist,
And there are a few others, who lodge in the Fist.
Carpus, Metacarpus, Phalanges, or Digits,
Are their sweet pretty names, but these are but midgits.
Then poor little Pollex, the joy of each child,
Of whose virtues and faults, Palmists don't draw it mild.
Now although my Lecture is almost done,
We must speak of the Skull, before we go home.
And in adult life, our head contains
Twenty-two bones, and sometimes brains.
The number is greater still in youth,
I'm telling you nothing but the truth.
In old age again, the numbers less,
Less trouble to sort, should they get in a mess.
Now the Nasal and Frontal bones come first,
Though should you get injured, these are not the worst.
But should a brick fall on Parietal, or Occipital,
Just take a Cab to the very best Hospital.
And kind Doctors there, if they see you're not shamming,
Will put you to rights, with a little Trepanning.
The Mandible bone is the last bone of all,
And it's none the less useful because it is small.
Now, perhaps, you are glad that the bones are all done,
As this makes a good finish to Lecture One.

E. M. C.

### Appointments.

Toxteth Park Workhouse Hospital.—Miss Rebecca Ellis, the recently-appointed Superintendent of Nurses at Toxteth Park Workhouse Infirmary, was trained at Kensington Infirmary, where she afterwards worked first as nurse and then for three years as Night Superintendent of Nurses. Miss Ellis was Home Sister at the Western Fever Hospital (Metropolitan Asylums Board) and Matron of the temporary Kensington Infirmary at Plaistow, Essex. Miss Ellis holds excellent testimonials, and we wish her every success.

Richmond, Whitworth, and Hardwicke Hospital, Ireland.—Miss Macdonnell, Superintendent of Nurses in the Richmond Hospital, has accepted the post of Lady Superintendent of the Richmond, Whitworth, and Hardwicke Hospitals.

Miller Hospital, Greenwich.—Miss Elizabeth Louise Underhill, lately Night Superintendent of Infirmary, East Dulwich, has been appointed Matron to the Miller Hospital, Greenwich.

### Where to Go.

St. Martin's Town Hall.—On Monday, March 5th at eight p.m., an evening entertainment will be given for the benefit of the Trained Nurses' Club, 12, Buckingham Street, Strand. There is an excellent programme, and tickets, 2s. 6d., 1s., and 6d. each, can be obtained at the club, or from Mrs. Gordon Shaw, 9, Barkston Gardens, S.W., or Miss Ethel Robinson, 95, Philbeach Gardens, S.W.

A lecture will be given at 13, York Place, Baker Street, by Mrs. Fawcett, at eight p.m. on Friday, March 9th. Subject: "Why Teachers should Interest Themselves in the Suffrage."

Trained Nurses' Club, 12, Buckingham Street, Strand. —Nursing Talks at 3 on Tuesdays. Lecture at 7.45, March 16th, by Dr. Oswald Browne. Subject, "The Care of the Dying."

London Obstetrical Society.—Next examination for diploma, second Wednesday in April.

### Everybody's Opinion.

[Correspondence on all subjects is invited, but we cannot in any way be responsible for the opinions expressed by our correspondents. No communications can be entertained if the name and address of the correspondent is not given, or unless one side of the paper only be written on.]

### CHARGE NURSES OR SISTERS?

"The Superintendent of a Training School" writes: I am trying to work a new system here for nursing the hospital' or rather it is an old one elsewhere, but new in these parts. At present we have permanent staff nurses and probationers —the former a great hindrance to the training of our own probationers. I therefore propose doing away with the permanent charge nurse and having only probationers and sisters, as I think it would make our training much better. I should like to know in what hospitals such a system has been found successful, and hope that some hospital readers will give me the benefit of their experience.

### BATH-ROOMS.

"A Country Nurse" writes: Referring to your paragraph in a recent issue of "The Nursing Mirror," I presume that you are alluding to hospitals where they have a ward kitchen, then articles like "jam and pickles" might be out of place in a bath-room. But what can one do when the bath-room and kitchen are together, as is the case here? The only place we have (for a male ward of 41 beds) is a room 14 feet by 8 feet, and in it the nurses have to wash soiled mackintoshes, dressing bowls, instruments, &c., and in the midst of all this the scrubber has her meals. You can imagine what it looks like about dinner time, when we have had several big dressings during the morning, and all the dinner things about. If you ever pay this hospital a visit, please do not judge the nurses' efficiency by the kitchen and bath-room combined, for you certrinly will see more than jam and pickles in ours.

### ATTENDANTS FOR THE INSANE.

"Edith Holmes" writes: In these days when so many gentlewomen are crowding into our hospitals and infirmaries to devote themselves to nursing, it seems strange, as it certainly is unfortunate, that so few have entered asylums to nurse the mentally afflicted. No doubt the work is hard, discouraging, monotonous, for results show slowly, and, in many cases, show none at all, while the experience is not so varied as it is in hospitals for the sick. The effect, however, on the patients would be highly beneficial. Women of intelligence and sympathy would more readily adjust themselves to the humane and liberal system used by the medical authorities at the head of these important institutions. The average attendant in asylums may be fairly intelligent and, more or less, in sympathy with her duties, but it is the exception to find among them sweet-voiced, gracious women who have higher ideas of life than its duties, who have quick intuitions for the needs of other women dependent on their care, and who have, too, some social qualities to share with their patients. In the case of those who are recovering, it is of incalculable benefit to be under the control and influence of such a nurse, to feel they have a friend always at hand, guiding and directing them day by day into the calm paths of reasonable conduct, watching with interest all efforts towards self-management, and, by advice and encouragement, fostering the slow and painful return to mental health. Kindness and patience are great aids to this result. A sensitive, shrinking woman, enervated by nervous prostration and exhausted by the double life she is leading—the life of the senses and the life of a perverted imagination, unhealthily excited or depressed—is aware, to some extent, of her surroundings, and is, in most cases, keenly conscious of the attitude of those round her towards herself. Unaware of what has led to the present position she may well be puzzled to understand why she is under the peremptory control of a hard-featured, harsh-voiced woman, who forces upon her rules and restrictions without any of that grace of manner which would make submission easy. The daily visits of the doctor make a pleasant break in this chilling, official routine, the sympathetic inquiry and kindly interest restore confidence, and show the importance of having large-minded, discriminating nurses who could extend these good effects. Restraint is, in many directions, absolutely necessary, but, where this is felt to be friendly, it is as reason returns, found to be judicious and curative. The nurses and attendants in asylums must hold this power over their patients, and how they use it is of vital consequence to them. Humour is a rare quality among women, but, in its place, some women are blessed with a cheeriness of disposition that can sparkle out into fun upon occasion. What a boon this is in an asylum where gloom is deepened by a matter-of-fact attendant, who has no margin, and never had, for the play of fancy, and in whom imagination is non-existent. In a country asylum one of the head attendants has done the same round of duty for fifteen years. She is still comparatively young, and would be pretty if her face did not wear an expression of chronic discontent. She "looks after" her patients with tired indifference, and, during intervals of her duties, sits back in a chair, throws her apron over her head, and (so the observer assumes) goes to sleep. She is not a stimulating presence, and, for her own benefit, requires a change of scene, and, probably of occupation. Asylum work may, in time, become depressing and exhausting if the worker is not on the outlook to take her own needed recreation in the way of rest and change. These charge attendants, by virtue of their position, give the tone to their subordinates. Generally young, and often heedless, they sometimes abuse their power, show an irritating want of respect for their patients, and may push along the feeble and the slow with quite unnecessary harshness. There are, happily, good-natured, kindly women among them who are interested in their charges, doing all they can for their comfort, and helping them towards recovery in every possible way. And there are, too, intelligent, companionable, cultured women, whose influence for good it would be difficult to over-estimate, and they make us wish their number increased in all hospitals for the insane.

### OLD-TIME NURSES.

"One Who Knows Their Difficulties" writes: It is with much pleasure I read your article on "Old-time Nurses." From personal experience I can fully sympathise with them, knowing the disadvantages under which they labour. I cannot understand why nurses who have had many years of hospital experience and responsibility should be considered by certain associations as not worthy to be placed by the side of nurses who have had, say, one quarter of the experience and not a tenth of the responsibility. I may mention as a case in point a well-educated woman, in the late thirties, with sixteen years' hospital experience, who has had charge of large surgical and medical wards, and has had charge of two cottage hospitals, applied lately to the R.B.N.A. to be registered as a trained nurse, and was refused, as her certificates of training, in the first place, were from a Women's Hospital. She was only a tenth of the time in that hospital, and has since had much experience in surgical, medical, and obstetric nursing. She was also refused admission to the Nurses' Co-operation on the same ground, viz., want of training. Sixteen years ago very few hospitals gave theoretical training, and in the provincial hospitals lectures were a thing unknown; but I think what was lost in theory was made up in practice. If one wished to get in one of the London training schools afterwards one was generally told that they did not train nurses who had been to other hospitals. All good wishes for the welfare of the old-time nurses, whose cause The Hospital has espoused.

### The Book World for Women and Nurses.

[We invite Correspondence, Criticism, Enquiries, and Notes on Books likely to interest Women and Nurses. Address, Editor, The Hospital (Nurses' Book World), 428, Strand, W.C.]

A Fair Claimant. By Frances Armstrong. (Blackie and Son, London, Glasgow, Edinburgh, and Dublin.)

"A Fair Claimant" is a story for girls. Stories for younger folk are becoming fewer and fewer nowadays, because the advanced younger generation demand to share the fiction intended for their elders, the doings of their contemporaries being less interesting to them. This is a pity, as some excellent writing has been devoted to books for the young. Frances Armstrong, in "A Fair Claimant," which is certain to be popular amongst youthful readers, has effected a compromise. She has cleverly given a story peopled with the older generation, full of stirring incident and quite harmless. It is full of those delightful improbabilities which fall in the apparently natural sequence, and abound in stories of adventure for boys, and which, to the young, looking at life as abounding in all manner of possibilities, has a peculiar fascination. In a new dress, the present tale narrates the life of that conventional heroine of fiction, the heiress lost in early life by her friends, and duly, after many adventures, coming into possession of her rightful inheritance. The heroine is not a particularly perfect or interesting young lady, but the defects of her personality will be lost sight of by youthful readers on account of the interesting episodes which surround her life.

The Queen at Balmoral. By Frank Pope Humphrey. (London: Fisher Unwin.)

Mr. Humphrey's book will be of great interest to those of the Queen's subjects who take a lively interest in her domestic life, and they, moreover, will be able to call their curiosity by the more euphonious title of loyalty. For we all like to think that it is loyalty, and not vulgar curiosity, which makes us wish to know how and where the Queen spends her days and what she says to her children and to her dependents. Balmoral was bought by Prince Consort with his own money, and is consequently the private property of the Queen. It is beautifully situated near the banks of the Dee, and Mr. Humphrey's description of its magnificent surroundings is exceedingly well and truthfully written. At first the house consisted of but few rooms; it has since been greatly added to, and both the outside and the inside of the present dwelling are fully described. It appears to be charmingly comfortable and home-like, and with none of the pomp and circumstance of Windsor or Buckingham Palace attached to it. We can well understand how the Queen, in the early days of her married life, loved to get to this solitude, away from all the constraint of Court life, and from "the bitterness which people create for themselves in London." We can fancy her delighting in the freedom of "running in and out all day, and going to chat with the old women." The foresters and cottagers have endless anecdotes of her kindly, courteous ways, and Mr. Humphrey has reproduced a goodly store of them. We are apt to forget, in these more common-sense days, that when the Queen was first married her household was arranged in a peculiarly uncomfortable and extravagant manner; and it is one of the many things for which we are indebted to the Prince Consort that put matters upon a better and more economical footing. Formerly the Lord Steward found the fuel for the palace fires, but the Lord Chamberlain had to have them lighted; the Lord Chamberlain provided the lamps, but the Lord Steward was answerable for the oil, and a window in the palace took many weeks to be either cleaned or mended, so much formal correspondence and red tape was necessary between the "Woods and Forests" who were accountable for the outside of the building, and the Lord Chamberlain, who presided over the inside; and the large staff of servants were almost without any control at all. Think then, home-loving reader, what a haven of rest, what a home, Balmoral must have always appeared to the Queen! She might order her own servants to do her bidding, and live in all ways as a rational woman as well as a queen! Years have gone by, and still the same walks and drives and cottage visiting go on, only now it is the grandchildren instead of the children that make the Highland home bright and gay, and who help to build the cairns on the neighbouring hill-tops when any especial event has to be commemorated.

Ophthalmic Nursing. By Sydney Stephenson, M.B., F.R.C.S.Edin., with sixty-one illustrations. Small 8vo., pp. 188. (London: The Scientific Press, Limited, 1894.)

Mr. Sydney Stephenson is very favourably known to the profession by the energy and skill with which he contended against an epidemic of contagious ophthalmia in the Hanwell Schools, and this little volume, which is mainly composed of his instructions to his nurses, will be exceedingly useful to those for whose benefit it is designed. It frequently happens that nurses otherwise well trained have not taken part in the actual work of eye wards, and hence are less useful to surgeons in ophthalmic practice than they might easily become. Reading can never form a thoroughly effectual substitute for clinical instruction, but it is at least far better than no instruction at all, and Mr. Stephenson has displayed an enviable power of condensing into small compass the most essential points of his subject. If we have any fault to find with his book it is that he enters somewhat too fully into the medical, as distinguished from the nursing, aspects of the questions of which he treats; but it is manifestly very difficult, if not impossible, to divide two closely-related subjects by a line which shall satisfy everybody. Nurses, at all events, will have no cause to complain of getting somewhat more information than the title of the book gives them a right to expect. Among minor blemishes, which should be removed in a second edition, we may point out that on p. 14, in the diagram intended to illustrate the position of the blind spot, either the cross and the black circle should change places, or the word "right" in the text should be "left," and *vice versâ*. On p. 30, "deny the impossibility" should be "deny the possibility." On p. 67, we read that "Dr. Tempest Anderson has made the excellent suggestion that ointments should be kept in collapsible lead tubes, like those used for oil paints"; and on p. 72, that "a new method of placing ointment in the eye has been brought under the notice of the profession by Dr. J. Ward Cousins, who advocates the use of a collapsible lead tube, the end of which is prolonged into a fine and flexible nozzle, &c." Perhaps it is meant that Dr. Anderson first suggested the use of the tubes for the preservation of the ointments, and Dr. Cousins for their application; but we were under the impression that such tubes were used in Paris long before they were known in England. At all events, the first examples which came under our notice were Parisian, and we shall be very glad to know that an Englishman, whether it be Dr. Anderson or Dr. Cousins, can claim priority for a useful and practical suggestion. Our last comment will be that the use of cocaine prior to operations, as described by Mr. Stephenson on page 78, would, in our experience, be insufficient to produce the total anæsthesia which is desirable for operations upon the eye; and that at least double the time should be devoted to the application, and double the quantity of the drug should be employed. With these few exceptions, we heartily commend Mr. Stephenson's book to the favourable notice of our readers.

